# Astrocyte plasticity shapes brain metastasis progression through tumor-astrocyte crosstalk

**DOI:** 10.47248/chp2502010002

**Published:** 2025-01-13

**Authors:** Rupleen Kaur, Nalin Gupta, Andrew Dhawan

**Affiliations:** 1. School of Medicine, Case Western Reserve University, Cleveland, OH 44106, USA;; 2. Rose Ella Burkhardt Brain Tumor and Neuro-Oncology Center, Cleveland Clinic, Cleveland, OH 44196, USA; 3. Biomedical Engineering, Washington University in St. Louis, MO 63130, USA;; 4. Cleveland Clinic Lerner College of Medicine, Case Western Reserve University, Cleveland, OH 44106, USA

**Keywords:** reprogrammed astrocytes, brain metastases, reactive astrocytes, tumor-associated astrocytes

## Abstract

Brain metastases are ten times more common than primary brain tumors and pose a significant clinical challenge. How brain metastatic tumor cells adapt to the unique and hostile brain microenvironment remains unclear. Astrocytes, the most abundant glial cells in the brain, are emerging as key mediators regulating the development of brain metastases. Initially anti-metastatic, astrocytes are reprogrammed by tumor-derived signals, transitioning into a pro-metastatic phenotype. Here, we review the roles of astrocytes in brain metastasis and describe the evidence for their phenotypic plasticity, the basis of astrocyte-tumor interactions, and potential therapeutic strategies targeting these processes.

## 1. Introduction

Brain metastases (BM) are the most common malignant intracranial tumors in adults, with over 100,000 new cases diagnosed annually in the United States [1]. These secondary tumors of the brain outnumber primary tumors tenfold, affecting 20–40% of all cancer patients [2,3]. The incidence of BM is increasing due to improved detection, more effective systemic and local therapies, and generally increased cancer incidence [4]. As such, BM are most commonly from primary lung cancers (30–60%), breast cancers (15–20%), skin cancers (5–10%), and gastrointestinal cancers (4–6%) [5]. Without treatment, survival is expectedly low and depends on the burden and degree of metastatic disease, as well as the primary tumor type [5]. However, recent advances in brain-penetrant chemotherapy, immunotherapy, and radiotherapy have led to increased progression-free and overall survival [6]. Patients with BM are most often treated with radiotherapy (whole brain radiation therapy or stereotactic radiosurgery) and chemotherapy, with surgery reserved for patients with singular accessible lesions or when a biopsy is needed [7]. Chemotherapy is generally felt to be less effective for the treatment of BM due to the difficulty in achieving therapeutic concentrations of drugs across the blood-brain barrier (BBB) and the development of chemoresistance [8].

The brain microenvironment presents distinct challenges to metastatic tumor cells with its unique cellular composition, metabolic features, and immune milieu. These characteristics impose strong selective pressure on tumor cells, shaping both the metastatic process and their response to treatment [9]. This microenvironmental influence on tumor progression is not exclusive to the brain. For instance, cancer-associated fibroblasts (CAFs) have drawn considerable attention for their role in promoting tumor growth and therapy resistance [10]. Arising from normal resident fibroblasts, CAFs are central to the remodeling of the extracellular matrix (ECM), immune modulation, and the secretion of pro-tumorigenic growth factors [11]. While most studies emphasize the tumor-promoting role of CAFs, recent research suggests that CAFs may also act to inhibit cancer progression, a contradiction explained by their high degrees of heterogeneity and plasticity.

Like CAFs, astrocytes in the brain play a critical role in maintaining homeostasis and display extensive transcriptomic heterogeneity and plasticity [12,13]. Similar to fibroblasts, astrocytes can display a pro-tumorigenic phenotype, promoting tumor progression and resistance to therapy [14]. In this review, we will explore the interactions between metastatic tumor cells and astrocytes in the brain microenvironment, focusing on mechanisms of astrocyte plasticity, key signaling pathways involved, and opportunities for therapeutic intervention. Although brain metastases arise from various primary tumors, in this review we take a tumor-agnostic approach, focusing instead on the common mechanisms of astrocyte plasticity and astrocyte-tumor interactions.

## 2. Role of Astrocytes in Normal Brain and Brain Injury

Astrocytes are essential for maintaining homeostasis, regulating the BBB, and supporting neuronal function [15]. Astrocytes regulate the blood-brain barrier by modulating the activity of alkaline phosphatase and the sodium-potassium ATPase on the endothelial cells of blood vessels [16]. Additionally, astrocytes mediate the transport of neutral amino acids via transporters in the System N family, including SNAT3, which is crucial for the uptake and release of glutamine—a precursor for glutamate, an excitatory neurotransmitter [17].

Following acute brain insult, such as traumatic brain injury or stroke, astrocytes transition into a reactive state — a process known as reactive astrogliosis (Figure 1) [15]. Reactive astrogliosis is marked by hypertrophy, increased expression of glial fibrillary acidic protein (GFAP), and enhanced proliferative activity [18]. *In vivo* models of traumatic brain injury have demonstrated upregulation of GFAP and morphological changes in astrocytes within hours following an injury [19]. Reactive astrocytes undergo morphological and functional transformations that allow them to manage debris and cordon off damaged areas of brain to prevent further injury spread [18]. This is facilitated by the formation of a glial scar, marked by the formation of a dense network of astrocytes at the injury site [20].

## 3. Astrocytes in Brain Metastases

Unlike acute brain injuries, brain metastases are a continuous, progressive insult to the central nervous system. BM are established when circulating tumor cells breach the BBB, a tightly regulated endothelium that restricts access to the brain [21]. Initially, reactive astrocytes act to prevent colonization by blocking cancer cells from crossing the BBB [14]. However, tumor cells disrupt the integrity of the BBB by secreting the enzymes cathepsin S and placental growth factor, which weaken cellular junctions, allowing tumor cells to infiltrate the brain parenchyma [22].

The majority of tumor cells that cross the BBB do not survive in the relatively harsh brain microenvironment [23,24]. The tumor cells that survive adhere closely to the surfaces of neural blood vessels in a process known as vascular co-option [24–26]. In response to vascular co-option and the breach of the BBB by tumor cells, reactive astrocytes initially act in an anti-metastatic manner, by secreting plasminogen activators (PA) [27]. PA convert plasminogen into plasmin, which then cleaves the Fas ligand, allowing for its diffusion which then results in apoptosis in brain metastatic cancer cells. Plasmin also has been shown to suppress brain metastases by inactivating the L1 cell adhesion molecule, which is crucial for vascular co-option and metastatic outgrowth [27].

Brain metastatic cells have been shown to counteract these antitumor mechanisms by increasing expression of anti-plasminogen activator serpins, such as neuroserpin and serpin B2 [27]. These serpins inhibit plasmin generation and therefore its anti-metastatic effects. Brain-metastatic lung and breast cancer cell lines upregulate the expression of these anti-PA serpins by more than threefold, effectively preventing plasmin-mediated apoptosis and promoting tumor cell survival within the brain microenvironment [27].

As brain metastases further develop, astrocytes unable to eliminate tumor cells can themselves become co-opted by cancer cells to become pro-tumorigenic (Figure 1). Klein *et al*. demonstrated this in a murine model of melanoma brain metastases, showing that metastatic lesions in the brains of affected mice were surrounded and infiltrated by astrocytes that showed a strong upregulation of the pro-inflammatory cytokine interleukin-23 (IL-23) [28]. IL-23 was shown to enhance melanoma cell invasiveness by upregulating matrix metalloproteinase 2 (MMP2) [28]. In addition to IL-23, these reprogrammed astrocytes secreted additional pro-tumorigenic proteins, including endothelin-1 (ET-1), hepatocyte growth factor (HGF), brain-derived neurotrophic factor (BDNF), interferon-alpha (IFN-α), and tumor necrosis factor-alpha (TNF-α) [29–32]. Thus, tumor-reprogrammed astrocytes *in vivo* displayed a marked shift in astrocyte behavior from an anti-tumorigenic role to a pro-tumorigenic role.

Further, there is a distinct difference between reactive astrocytes in brain injury — which exhibit a proliferative and inflammatory phenotype — and brain metastasis-associated (BM-associated) astrocytes, which have been reprogrammed by tumor cells to be pro-tumorigenic. Reprogrammed astrocytes actively support the growth and development of brain metastases, aiding tumor cells in adapting to their new habitat.

## 4. Key Signaling Pathways Involved in Astrocyte Reprogramming and Crosstalk with Tumor Cells

The tumor-mediated reprogramming of BM-associated astrocytes is orchestrated by a complex crosstalk between tumor cells and astrocytes, involving a network of signaling pathways, cytokines, growth factors, and extracellular vesicles. The following sections will explore the key pathways and mechanisms involved.

### 4.1 STAT3 pathway activation

Priego and colleagues, in a study of 91 resected human metastases, identified a subpopulation of astrocytes proximal to brain metastases, characterized by nuclear-localized STAT3 activation. STAT3 activation was shown to promote metastatic growth by modulating the innate and acquired immune system [33]. Specifically, astrocytes with activated STAT3 suppressed CD8+ T cell activation and recruited immunosuppressive CD74+ microglia/macrophages around the tumor, creating an immune-suppressed environment supporting tumor survival (Figure 2). Conditional deletion of STAT3 in reactive astrocytes in a murine model led to a significant reduction in brain metastasis [33].

STAT3 (signal transducer and activator of transcription-3) is a transcription factor and intracellular signaling protein that regulates critical biological processes such as cell differentiation, proliferation, and apoptosis [34]. In the context of brain metastasis, STAT3 activation promotes a shift in astrocytes from their normal role in neural homeostasis to a pro-tumorigenic phenotype. Priego *et al*. observed that STAT3 activation in astrocytes is driven by various factors secreted by tumor cells, such as interleukin-6 (IL-6), epidermal growth factor (EGF), transforming growth factor-alpha (TGF-α), and ciliary neurotrophic factor (CNTF) (Figure 2) [33]. These tumor-secreted factors bind to receptors on astrocytes, triggering the activation of Janus kinases (JAKs). Once activated, JAKs phosphorylate STAT3, which then dimerizes and translocates to the nucleus, where it acts as a transcription factor, promoting the expression of genes that enhance cell survival, proliferation, and immune evasion [34,35].

Further, using overall survival from diagnosis for 39 patients with brain metastases originating from various primary tumors, Priego *et al*. demonstrated that patients with higher levels of activated STAT3 in the tumor microenvironment had significantly poorer clinical outcomes compared to those with lower STAT3 levels (*p* = 0.0116) [35].

### 4.2 Notch signaling

The Notch signaling pathway is essential for normal development and plays a critical role in regulating cell fate decisions, including the self-renewal of adult stem cells and the differentiation of progenitor cells into specific lineages [36]. Xing *et al*. (2013) demonstrated that conditioned media from brain-metastatic breast cancer cell lines significantly upregulated the Notch ligand Jagged-1 (JAG1) at both the mRNA and protein levels in primary rat and immortalized human astrocytes, while other Notch ligands such as JAG2, DLL1, DLL3, or DLL4 were unaffected [37]. This specific increase in JAG1 expression was shown to result from NF-κB pathway activation, driven by interleukin-1 beta (IL-1β) secreted at high levels by brain-metastatic tumor cells [37]. The binding of astrocyte-expressed JAG1 to the Notch receptor on tumor cells initiates Notch signaling in the brain metastatic tumor cells (Figure 2) [38]. Upon binding JAG1, the Notch receptor undergoes cleavage by gamma-secretase, releasing the Notch intracellular domain (NICD), which translocates into the nucleus [37,39]. There, NICD forms a complex with CSL/RBP-J, upregulating transcription of HES5, a factor that enhances stemness, survival, and chemoresistance in tumor cells (Figure 2) [37]. This aberrant activation of Notch signaling reinforces the stem-like properties of CSCs, driving their invasiveness and resistance to therapies. Unlike other signaling pathways that rely on kinase cascades for amplification, Notch signaling is uniquely sensitive to signal intensity [37,40]. The amount of NICD in the nucleus directly influences the strength and duration of the transcriptional response, shaping tumor aggressiveness [40].

### 4.3 Matrix metalloproteinases

Matrix metalloproteinases (MMPs), a family of zinc-dependent proteases, are critical for degrading components of the extracellular matrix (ECM), such as collagen and laminin [41]. Under normal physiological conditions, MMP activity is tightly controlled to preserve tissue structure and support repair processes. However, in the tumor microenvironment, this regulation is disrupted, resulting in excessive ECM degradation that facilitates tumor progression. Studies in brain metastasis models of melanoma have demonstrated that tumor cells induce reactive astrocytes to secrete the inflammatory cytokine IL-23, which in turn stimulates the expression of matrix metalloproteinases MMP2 and MMP1 via the NF-κB signaling pathway [42,43]. The dysregulated MMP expression results in excessive ECM degradation, enabling tumor cells to infiltrate the brain parenchyma. MMP2 has been associated with increased tumor invasiveness, the formation of a pro-metastatic niche, and poor patient outcomes [42,43]. Likewise, MMP1 has been shown to alter blood-brain barrier (BBB) permeability by degrading key tight junction proteins, such as claudin and occludin, allowing metastatic cells to further infiltrate the brain [42,43].

Beyond the NF-κB pathway, MMP2 expression is also regulated by the ERK1/2 (extracellular signal-regulated kinase) pathway [44]. Astrocyte-secreted factors, such as PGE2, activate ERK1/2 in cancer cells via ERK1/2 phosphorylation, leading to further upregulation of MMP2 transcription [44]. Astrocyte-conditioned media has also been shown to induce ERK1/2 activation in brain metastatic cells, resulting in increased MMP2 expression and enhanced tumor cell invasion into the brain [44]. Additionally, MMP1, driven by COX2 expression in brain metastatic cells, upregulates CCL7 secretion in astrocytes [45]. CCL7 release stimulates a positive feedback loop, wherein tumor cells further activate astrocytes to maintain a pro-metastatic environment, particularly through the renewal of tumor-initiating cells by Nanog expression [45].

The breakdown of the ECM by MMPs not only facilitates tumor invasion but also liberates growth factors such as vascular endothelial growth factor (VEGF), which promotes angiogenesis and supports tumor expansion (Figure 2) [46]. Moreover, MMPs may modulate the immune response by cleaving cell surface molecules and cytokines, enabling tumor cells to evade immune detection and destruction.

### 4.4 Exosomes

Exosomes are small membrane-bound vesicles secreted by cells, carrying a range of biomolecules such as proteins, lipids, and genetic material, including DNA, mRNA, and miRNA. Exosomes serve as mediators of communication between tumor cells and astrocytes within the brain microenvironment. Through the transport of molecular cargo, exosomes enable intercellular signaling that can modulate cellular state in the local microenvironment [47]. Tumor-derived exosomes can induce reactive astrocyte reprogramming, leading to changes in their secretory profile that support tumor growth. For example, *in vitro* experiments co-culturing H1299-derived exosomes with SVG P12 astrocytes demonstrated significant changes in cytokine release [48]. Specifically, the exosome-treated astrocytes upregulated pro-tumorigenic cytokines such as GROα/CXCL1, IFN-γ, VEGF, and additional factors such as IL-3, IL-5, and M-CSF, while downregulating IL-7 (Figure 2) [48]. These changes suggest a marked shift toward a reactive, pro-tumorigenic state that creates a supportive microenvironment for metastatic cells.

In a positive feedback loop, reprogrammed astrocytes that secrete exosomes induce further tumor cell co-option of resident neural cells. Zhang *et al*. showed that astrocyte-derived exosomes were found to contain and transfer miR-19a to tumor cells, leading to downregulation of *PTEN* (phosphatase and tensin homolog), a known tumor suppressor gene (Figure 2) [49]. The loss of *PTEN* expression in these tumor cells resulted in the activation of the NF-κB signaling pathway, which subsequently upregulated the secretion of the chemokine CCL2 [49]. CCL2 has been shown in murine models of brain-metastatic breast cancer to play a critical role in recruiting IBA1+ myeloid cells, such as macrophages and microglia, to the brain metastatic site, further acting to promote brain metastasis (Figure 2) [49].

### 4.5 Hyaluronic acid

Tumor-associated astrocytes produce elevated levels of hyaluronic acid (HA), which interacts with various extracellular matrix (ECM) receptors, including hyaluronan-mediated motility receptors (HMMR) [50]. HMMR are expressed at high levels in tumor cells and are central to promoting micrometastatic outgrowth. By activating pathways such as ERK/MAPK, HMMR increases cell motility, facilitates ECM degradation, and drives metastatic progression. While HMMR is not required for the initial seeding of tumor cells in the brain, it plays a crucial role in the expansion of micrometastases [50]. Knockdown of HMMR in tumor cells significantly reduces cancer cell cluster formation and migration [50]. Co-culturing tumor cells with HA-producing reactive astrocytes further enhances the invasive phenotype of these cells, as measured by organoid outgrowth quantified through tumor cell luciferase activity [50]. HMMR-mediated ECM degradation also releases growth factors such as VEGF, which promote angiogenesis and support tumor expansion [51].

## 5. Role of Gap Junctions in Tumor-Astrocyte Interactions

Brain metastatic tumor cells may exhibit increased expression of protocadherin 7 (PCDH7) [52]. PCDH7 facilitates the preferential formation of gap junctions between cancer cells and astrocytes, with these junctions being composed primarily of Cx43 [32]. Through these connections, secondary messengers such as cGAMP are transferred from tumor cells to astrocytes, activating the STING pathway, an innate immune response pathway to cytosolic double-stranded DNA [32]. This stimulates astrocytes to produce inflammatory cytokines such as IFNα and TNFα. These cytokines, in turn, activate the STAT1 and NF-κB signaling pathways in tumor cells [32]. These activated pathways promote cancer growth, enhance resistance to external stressors, and drive further metastatic expansion.

One major consequence of astrocyte-tumor gap junctions is the induction of therapeutic resistance. Lin *et al*. demonstrated that co-culturing reactive astrocytes with human brain metastatic melanoma cell line TXM-13 reduced chemotherapy-induced apoptosis by 70% [53]. This protective effect was not specific to the chemotherapeutic agent as similar results were observed using cytotoxic drugs such as cisplatin and paclitaxel [53]. The protective effect is attributed to the formation of Cx43 gap junction complexes, which facilitate calcium transfer between astrocytes and tumor cells. When melanoma cells were exposed to chemotherapy without astrocytes, increased cytoplasmic Ca^2+^ levels induced apoptosis. However, co-culturing with astrocytes significantly reduced Ca^2+^ levels in the tumor cells, suggesting that astrocytes shield tumor cells from chemotherapy by sequestering calcium via the Cx43 gap junctions [53].

Additional studies have corroborated this resistance to chemotherapy through the activation of alternative signaling pathways. Specifically, co-culturing tumor cells with astrocytes is protective from taxol-induced apoptosis, via upregulation of interleukin 6 (IL-6) and interleukin 8 (IL-8) [54]. IL-6 and IL-8 stimulate astrocytes to produce endothelin-1 (ET-1), which subsequently increases the expression of endothelin receptor A (ETAR) and endothelin receptor B (ETBR) on cancer cells [54]. Activation of ETAR and ETBR promotes the expression of survival genes such as BCL2L1, GSTA5, and TWIST1 via the AKT/MAPK pathway, further protecting cancer cells from taxol-induced apoptosis [54].

## 6. Therapeutic Implications of Astrocyte Reprogramming

Targeting the interactions between tumor cells and astrocytes presents a significant biological challenge. The blood-brain barrier (BBB) remains a major obstacle in treating brain metastases [8]. Astrocytes, which form the outer layer of the BBB through their end feet, express glucose transporter proteins that actively pump drugs and neuroactive compounds out of the brain [55]. This limits the ability of therapeutic agents to reach the tumor microenvironment. Even when drugs are able to cross the BBB and enter the tumor microenvironment, astrocyte-tumor interactions via direct communication (*i.e*., gap junctions), may persist.

Astrocyte heterogeneity further complicates the design of TME-based therapeutic strategies. Astrocytes exhibit substantial heterogeneity in their expression of marker proteins, such as glial fibrillary acidic protein (GFAP) and excitatory amino acid transporter 1 (EAAT1/GLAST) [26]. Single-cell mRNA sequencing has revealed significant transcriptional heterogeneity among astrocytes, both across different brain regions and within the same region [56]. This transcriptional diversity poses a challenge for identifying and targeting specific astrocyte-tumor interactions.

Despite these challenges, several potential therapeutic targets have been identified (Table 1). Inhibitors targeting the key aforementioned pathways, including STAT3, Notch, and NF-κB, are currently being tested in clinical trials. Astrocyte-directed antitumor therapies must have a target that is specific to reprogrammed astrocytes, thereby sparing normal brain tissue, and must be penetrant to the blood-brain barrier.

## 7. Analogies to Cancer-Associated Fibroblasts

Fibroblasts are interstitial mesenchymal cells responsible for synthesizing collagen and maintaining the structural integrity of tissues. These cells, like astrocytes, exhibit substantial phenotypic and functional heterogeneity across different tissues and conditions. When activated within the tumor microenvironment, fibroblasts transform into cancer-associated fibroblasts (CAFs), which promote tumor progression through extracellular matrix (ECM) remodeling, immune evasion, and paracrine signaling (Figure 3) [70]. As with reprogrammed astrocytes, CAFs engage in complex, bidirectional communication with tumor cells by secreting growth factors and cytokines such as HGF, VEGF, and TGF-β (Figure 3). CAFs also contribute to ECM remodeling by producing matrix metalloproteinases (MMPs) [70].

To date, various subtypes of CAFs have been identified, including myofibroblastic, inflammatory, and antigen-presenting CAFs [71]. However, due to the plasticity of CAF phenotypes, these classifications can be somewhat misleading. This heterogeneity mirrors that of astrocytes, where no single marker can sufficiently identify all tumor-supportive astrocytes. Moreover, despite advances in understanding CAFs, therapeutic strategies targeting CAFs have yielded mixed results [72]. Some therapies have focused on disrupting the signaling pathways that drive CAF reprogramming—such as the TGF-β/SMAD and NF-κB pathways—but the inherent heterogeneity and plasticity of CAFs have made it difficult to achieve consistent clinical success [73]. This highlights the need for therapies that can selectively target CAF subtypes.

Astrocyte-targeted therapies face challenges analogous to those of CAF-targeted therapies. Given astrocytes’ vital roles in the central nervous system, any therapeutic approach must balance disrupting tumor-supportive signaling and preserving the essential functions of astrocytes. Like CAFs, astrocytes exhibit significant heterogeneity and plasticity, and their roles in brain metastases evolve as tumors progress [56]. Better defining astrocyte subpopulations in both healthy brain tissue and tumors will be essential for identifying therapeutic targets and developing more precise and effective treatments.

## 8. Conclusions

Brain metastases, primarily originating from lung cancer, breast cancer, and melanoma, outnumber primary malignancies tenfold. For metastatic tumor cells to colonize the brain, the cells must overcome the selective pressure of the blood-brain barrier and adapt to the unique brain microenvironment. Upon breaching the blood-brain barrier, metastatic cells encounter a challenging and plastic environment, which is ultimately transformed into a tumor-promoting microenvironment. Astrocytes, one of the most abundant cell types in the brain, are a key component of this transformation, as there is emerging evidence for their plasticity mediated by tumor cells to transition into a pro-metastatic phenotype. Tumor cells co-opt astrocytes by hijacking key signaling pathways and altering immune responses, converting astrocytes into facilitators of metastasis. This hijacking leads to increased proliferation of tumor cells and therapeutic resistance.

The complexity of astrocyte-tumor cell interactions, compounded by the heterogeneous distribution and varied functions of astrocytes across different brain regions, presents significant challenges for developing targeted therapies aimed at reversing or halting astrocyte reprogramming. Future work will focus on elaborating a deeper understanding of the molecular and cellular mechanisms underlying astrocyte reprogramming.

## Figures and Tables

**Figure 1.**
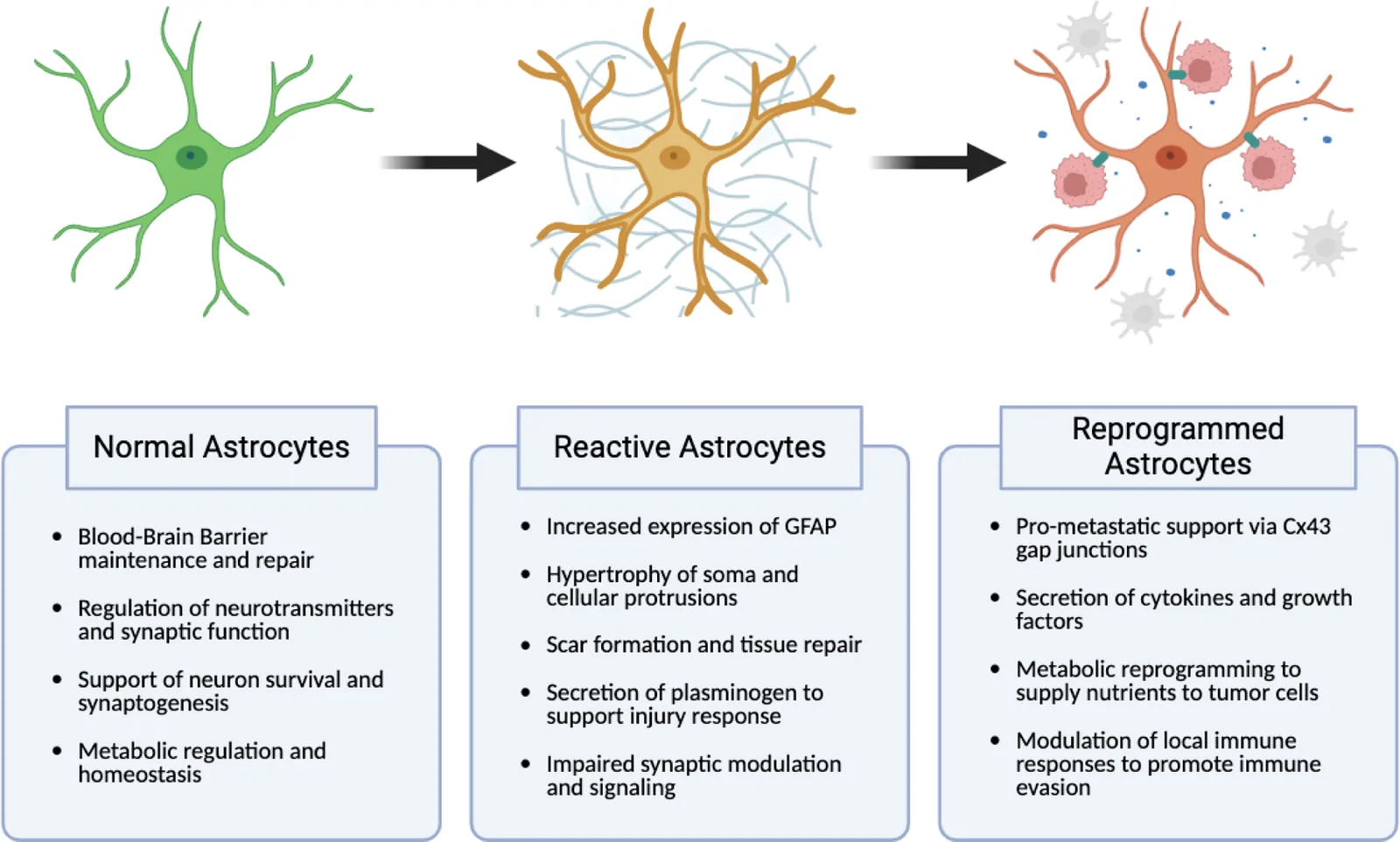
Progression of tumor-associated astrocytes from normal to reprogrammed. Astrocytes, encountering tumor cells, enter a reactive state characterized by hypertrophy, increased GFAP expression, and anti-metastatic properties (e.g., plasminogen secretion). Over time, reactive astrocytes undergo further reprogramming by tumor-secreted factors, such as cytokines and growth factors, that drives them toward a pro-metastatic, reprogrammed phenotype [14].

**Figure 2.**
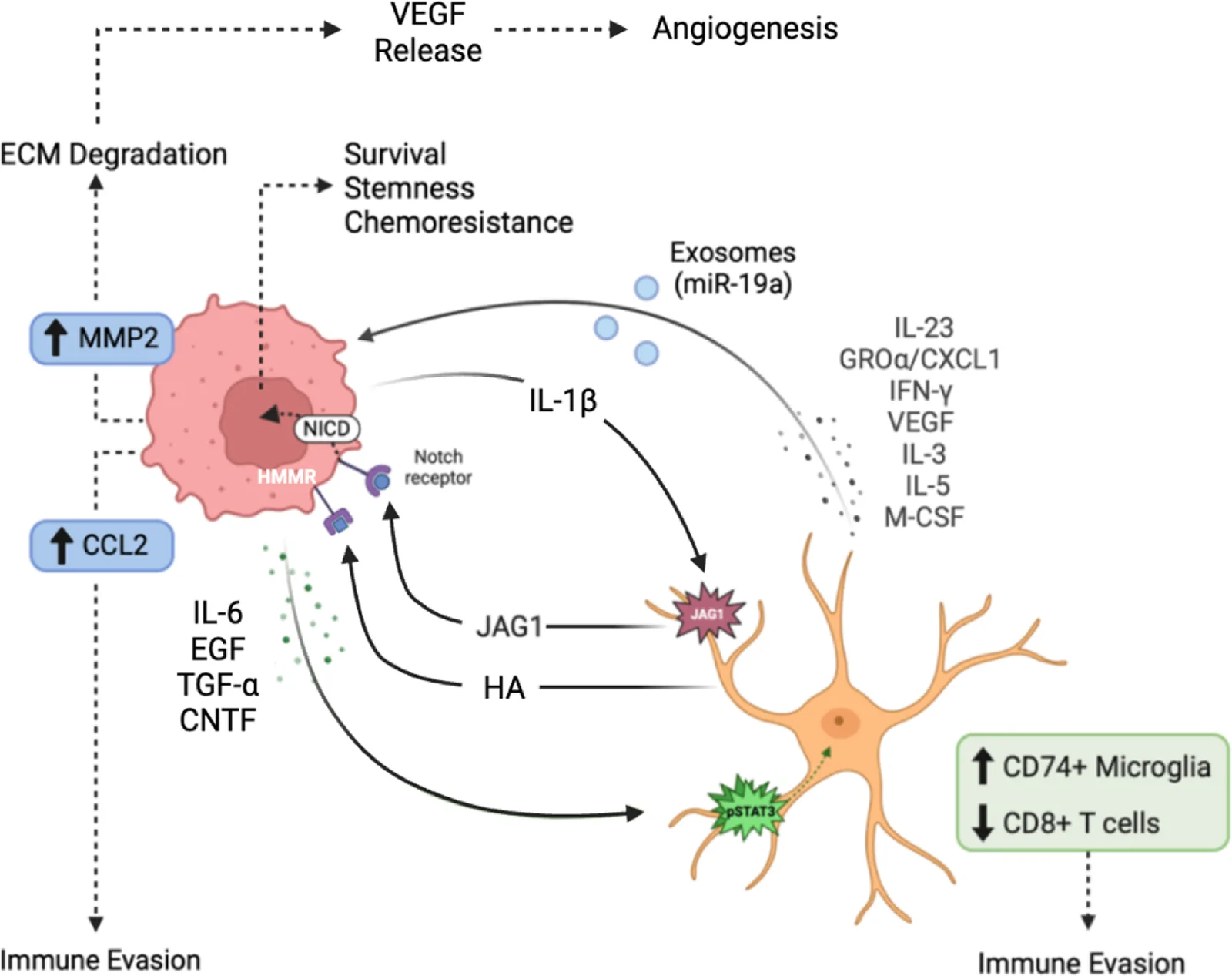
Key Signaling Mechanisms of Astrocyte Reprogramming in the Tumor Microenvironment. Tumor-secreted factors induce astrocyte reprogramming into a pro-metastatic state, characterized by STAT3 and Notch signaling activation. Reprogrammed astrocytes promote tumor progression through immune module and paracrine signaling. This dynamic crosstalk between tumor cells and reprogrammed astrocytes establishes a microenvironment conducive to metastasis and tumor growth.

**Figure 3.**
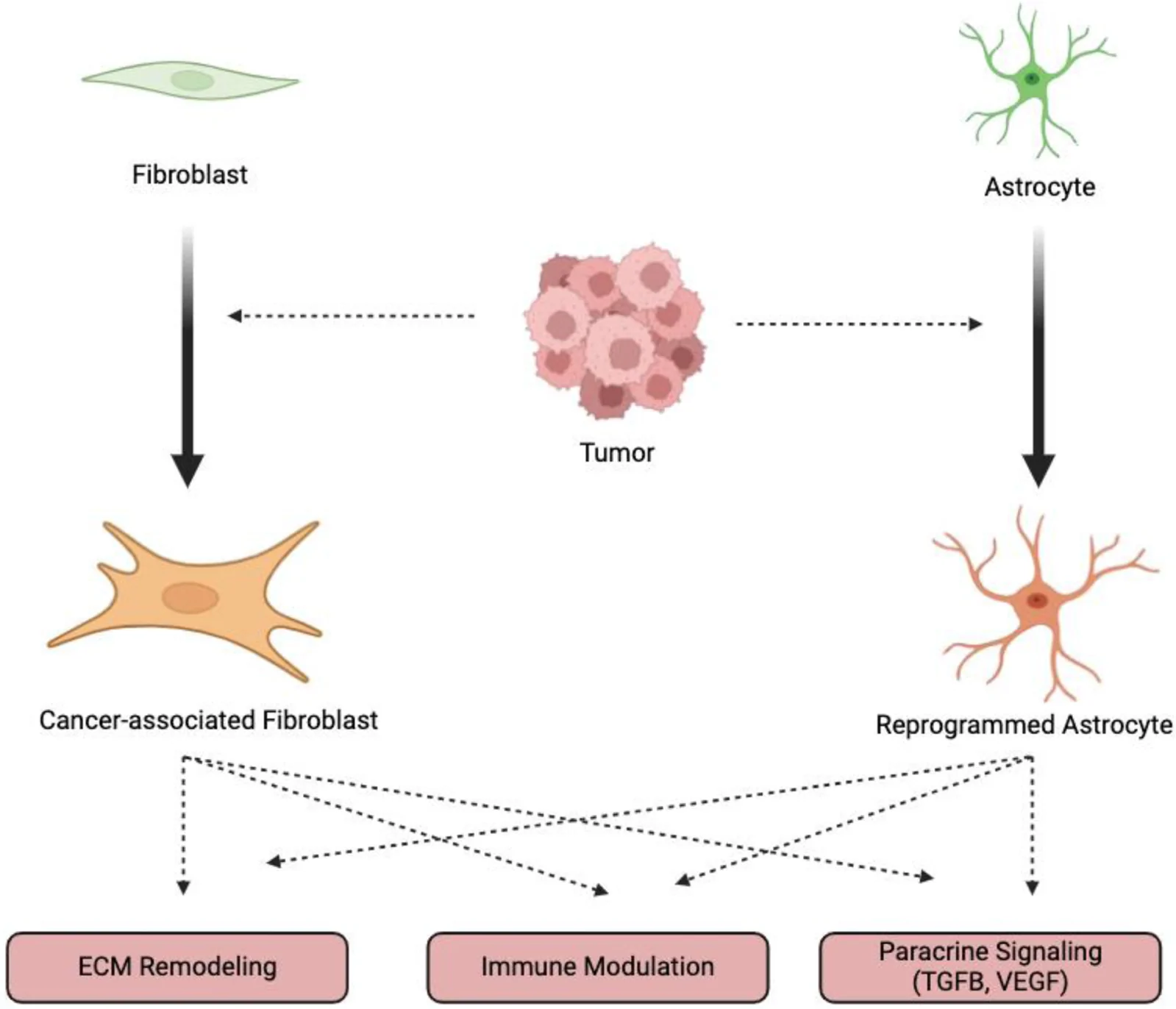
Analogies between cancer-associated Fibroblasts (CAFs) and reprogrammed astrocytes in the tumor microenvironment. Fibroblasts and astrocytes, upon exposure to tumor-derived factors, undergo reprogramming within the tumor microenvironment to transition into cancer-associated fibroblasts (CAFs) and reprogrammed astrocytes, respectively. Both cell types contribute to tumor progression through extracellular matrix (ECM) remodeling, immune modulation, and paracrine signaling.

**Table 1.** Therapeutic Agents Targeting Reprogrammed Astrocytes in Brain Metastasis.

Drug Name	Molecular Target	Therapeutic Effect	References
Macitentan	Endothelin Receptor A and B	Macitentan reduces brain metastasis and tumor burden when combined with paclitaxel. It has shown efficacy in lung and breast cancer brain metastasis models. It targets astrocytes and endothelial cells, reducing pAKT and pMAPK levels, which contributes to reduced metastasis.	[57–59]
Meclofenamate	Cx43 Gap Junctions	Meclofenamate disrupts gap junction mediated communication between astrocytes and cancer cells. It has shown to reduce lung and breast cancer brain metastases. It also renders brain metastatic melanoma cells more sensitive to chemotherapeutic drugs such as paclitaxel, cisplatin, and 5-FU. Its effects are specific to brain tissue and do not influence primary tumors in the breast or lung.	[60–63]
Tonabersat	Cx43 Gap Junctions	Tonabersat, similar to meclofenamate, disrupts Cx43-mediated gap junction communication. This disruption has shown to reduce tumor burden in brain tissue models of metastasis. Its efficacy is specific to brain metastases, with no observed impact on primary tumors.	[60–63]
Pazopanib	VEGFR1-3, PDGFRα-β, c-kit, B-Raf	Pazopanib, a multi-target tyrosine kinase inhibitor, has been correlated with reducing the proliferative capacity of immortalized astrocyte cell lines *in vitro*. It has demonstrated efficacy in preventing brain metastasis in HER2+ brain metastasis models.	[64,65]
Compound E	JAG1-Notch	Compound E is a gamma-secretase inhibitor that blocks JAG1-Notch signaling, resulting in reduced HES5 expression. It has been shown to suppress the cancer stem-like phenotype in brain metastasis models and significantly decreases the growth of brain metastatic breast cancer cells *in vivo*.	[37]
Lenalidomide	FN14	Lenalidomide disrupts FN14/TWEAK signaling, reducing astrocyte reactivity. Its primary application is in treating inflammatory CNS conditions like multiple sclerosis.	[66–68]
Legasil	STAT3	Legasil, containing silibinin, inhibits STAT3 activation in reactive astrocytes. It has demonstrated significant clinical and radiological improvements in patients with brain metastases from non-small-cell lung cancer who progressed after whole-brain radiotherapy and chemotherapy.	[69]

## Abbreviations

BBB: Blood-brain barrier
BM: Brain metastases
CAFs: Cancer-associated fibroblasts
CCL2: Chemokine (C-C motif) ligand 2
CSCs: Cancer stem cells
ECM: Extracellular matrix
ET-1: Endothelin-1
FN14: Fibroblast growth factor-inducible 14
GFAP: Glial fibrillary acidic protein
HA: Hyaluronic acid
HMMR: Hyaluronan-mediated motility receptors
JAG1: Jagged-1
JAK: Janus kinases
MMP: Matrix metalloproteinase
NICD: Notch intracellular domain
STAT3: Signal transducer and activator of transcription-3
STING: Stimulator of interferon genes
TWEAK: Tumor necrosis factor-like weak inducer of apoptosis
